# Electronic Health Records That Support Health Professional Reflective Practice: a Missed Opportunity in Digital Health

**DOI:** 10.1007/s41666-022-00123-0

**Published:** 2023-01-20

**Authors:** Anna Janssen, Judy Kay, Stella Talic, Martin Pusic, Robert J. Birnbaum, Rodrigo Cavalcanti, Dragan Gasevic, Tim Shaw

**Affiliations:** 1 Faculty of Medicine and Health, The University of Sydney, Level 2, Charles Perkins Centre D17, Sydney, NSW Australia; 2 Faculty of Engineering, The University of Sydney, Sydney, Australia; 3 School of Public Health and Preventive Medicine, Monash University, Melbourne, VIC Australia; 4 Department of Department of Pediatrics, Harvard Medical School, Boston, MA USA; 5 Mass General Brigham, Harvard Medical School, Boston, MA USA; 6 Department of Medicine, University of Toronto, Toronto, ON Canada; 7 HoPingKong Centre, University Heath Network, Toronto, ON Canada; 8 Faculty of Information Technology, Department of Human-Centred Computing and Centre for Learning Analytics at Monash, Monash University, Clayton, VIC Australia

**Keywords:** Digital health, Electronic health records, Health informatics, User experience design

## Abstract

A foundational component of digital health involves collecting and leveraging electronic health data to improve health and wellbeing. One of the central technologies for collecting these data are electronic health records (EHRs). In this commentary, the authors explore intersection between digital health and data-driven reflective practice that is described, including an overview of the role of EHRs underpinning technology innovation in healthcare. Subsequently, they argue that EHRs are a rich but under-utilised source of information on the performance of health professionals and healthcare teams that could be harnessed to support reflective practice and behaviour change. EHRs currently act as systems of data collection, not systems of data engagement and reflection by end users such as health professionals and healthcare organisations. Further consideration should be given to supporting reflective practice by health professionals in the design of EHRs and other clinical information systems.

## Introduction

Electronic health records (EHRs) are repositories of patient health information, created by health professionals to capture data related to specific clinical encounters [1]. This type of information has great potential to enable health professionals to understand the quality of their performance and to support reflective practice and learning. Reflective practice is the process of revising habits, routines, and experiences to understand complex problems and learn from past behaviours [2, 3]. It is a process that can be engaged in by both individuals and groups of people [2]. In the context of the health workforce, reflective practice has been identified as an essential process for health professionals to maintain up to date knowledge and skills [4, 5].

To date, the potential of EHRs to support this has not been fully reached. The design of most modern EHR systems does little to encourage or enable health professional learning. Addressing this issue is important now because there has been a growing movement to make health information systems such as EHRs more interoperable [6]. Increased interoperability will enable a readily available flow of data that can be used for reflective practice by health professionals [6]. Furthermore, EHR development is currently undergoing an unprecedented era of innovation [7–9]. This is resulting in disruption to enterprise vendors by more agile next-generation EHRs that can respond to diverse needs of end users [10]. Innovation in EHR development runs parallel to similar technological innovations occurring in the education space such as the use of virtual and augmented reality [11] and bots [12] to transform learning. Finally, there are currently a range of changes in the policy landscape regarding how health professionals engage in learning activities; these emphasise the need for health professionals to use electronic data to understand their outcomes as a component of their mandated training activities and revalidation [13, 14]. In this commentary, we contextualise the intersection between digital health and data-driven reflective practice. Furthermore, we argue that EHRs are a rich source of information on the performance of health professionals and healthcare teams that could be harnessed to support reflection and learning.

Digital health is a diverse field that has been defined and redefined many times, but can be described as the use of electronic health data and digital technology to inform medical practice and improve health [15]. It includes consumer technologies such as apps and devices, communication technologies such as telehealth, and informatics technologies for capturing and harnessing electronic data [16]. Digital health also brings together multidisciplinary experts [17] to explore how technology can strengthen and transform healthcare. As a field, digital health includes individuals with expertise in human computer interaction, who seek to better understand the design of health technologies [18, 19], implementation scientists exploring how to implement and de-implement technologies [20], empirical researchers evaluating whether health technology improves health outcomes [21], and many other diverse stakeholders.

## Electronic Health Data

A foundational component of digital health involves collecting and leveraging electronic health data to improve health and wellbeing. EHRs are core technologies for collecting these data. EHRs are information systems used by healthcare organisations around the world to capture data on patient care. In the context of digital health, the literature has explored EHR technology from many perspectives such as:

- Investigating the knowledge and skills the health workforce require to utilise EHRs and the data within them [16, 22];
- Understanding how organisations can efficiently implement EHRs [23] and their use as signifiers of digital maturity [24];
- Evaluating the use of EHRs to undertake research that is integrated with routine clinical care [25];
- Investigating the effectiveness of the use of EHRs as tools for improving quality of care [26, 27] and predicting health trajectories [28];
- Exploring the potential of EHRs to support virtual care and other new healthcare models [29] and many applications beyond these.

The potential of EHR data for supporting tailored interventions to improve the processes and outcomes of care is gaining recognition by a range of disciplines in the health sector including medicine, nursing and allied health [30]. To date, the value of EHR data to support reflective practice via individual and team learning has been largely unrealised. This is surprising for a number of reasons. Firstly, and potentially most importantly, health professionals want to use EHR data for reflective practice and learning [30]. Secondly, many health professionals dedicate considerable work time in a range of learning activities [31]. Thirdly, reflection and workplace learning are recognised mechanisms to ensure health professionals stay up to date with emerging clinical evidence and best practice guidelines [32]. Finally, patients are increasingly advocating for increased involvement in collaborative clinical decision through data collection mechanisms such as patient reported measures and EHR gateways [33].

The first EHRs were developed in the 1970s [34], but the technology was not widely adopted until the late 1990s. The World Health Organization’s 2016 global survey of eHealth found that more than 50% (*n* = 23) of upper- and middle-income countries, 35% (*n* = 10) of lower-middle income countries and 10% (*n* = 3) of low-income countries have adopted national electronic records [35]. These data indicate there is an emerging trend in adopting EHRs in upper- and middle-income countries. EHRs are championed as a digital health solution that can offer wide reaching benefits at the clinical, organisational and societal level [36]. Improvements that EHRs can offer at the clinical level include minimisation of medical errors and improvements in care coordination [37].

The potential of EHRs for quality improvement [38], clinical research [39], professional development and practice improvement by health professionals [40] has already been recognised in the literature. Key barriers to using EHRs for these purposes include the perception that EHRs increase the workload of health professionals, particularly when the information systems are initially introduced [41]. Coupled with this, the usability and interoperability of EHRs have been recognised as a significant barrier to their adoption by the health workforce [42].

**Fig. 1:**
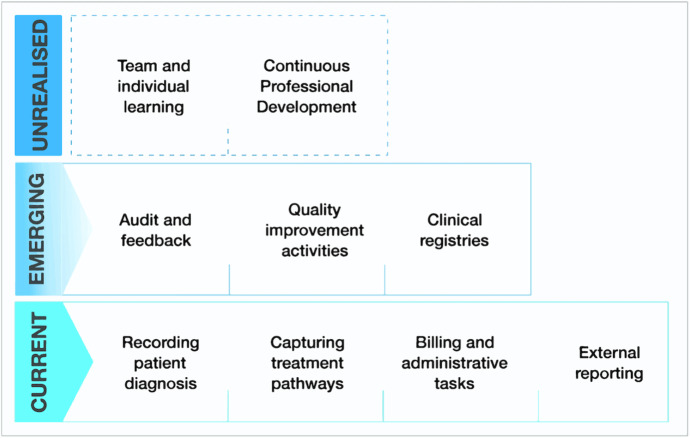
Characterisation of the main uses of EHR data in healthcare organisations, with current uses at the bottom, emerging trends in use in the middle, and, at the top, potential uses that are yet to be realised

Although there is now a growing interest in open-source EHRs, healthcare organisations still largely adopt costly proprietary information systems developed by enterprise vendors [1, 38]. The design of modern EHRs is still heavily informed by their legacy as billing and administrative information systems, which includes a general repository for patient data [37] and coding of patient data for administrative and billing purposes (Fig. 1) [1].

There is a considerable variation in the functionality of modern EHRs at different organisations around the world. At a minimum, most EHRs provide three key medical functions: (1) presenting the most-up-to date information on a patient, (2) storing supporting documentation about a patient (such as test results and imaging) and (3) enabling health professionals to input data about a patient’s diagnosis and treatment [1]. For the most part, modern EHRs act as systems of data collection, not systems of data engagement and reflective practice by end users such as health professionals and healthcare organisations.

## Harnessing Electronic Health Records for Reflective Practice

Critiques of EHR design have highlighted flaws that hinder their use for professional development and reflection. Of particular note is the criticism that the design of many EHRs creates undesirable cognitive burdens. These burdens include information overload, problems of situational awareness due to fragmented presentation of data and increasing cognitive demands through actions such as having to click through multiple screens to navigate systems [43]. Furthermore, EHR design does not account for the nature of collaborative clinical work and can therefore be particularly poor at supporting team-cognition [43]. These design problems may be exacerbated by weaknesses in user testing to evaluate technology interfaces. Such testing often fails to go beyond studies with individual health professionals. Yet these technologies are frequently used by healthcare teams; teams typically have different usability needs from individual practitioners. A recent review of EHR design identified the following common usability issues: naturalness, consistency, preventing errors, minimising cognitive load, efficient interaction, forgiveness and feedback, effective use of language, effective information presentation and customisation/flexibility [44]. Guidelines for addressing cognitive load seem particularly important for supporting reflective practice. Specifically, these include the guidelines for (a) the design of the EHR interfaces include minimising users’ mental workload; (b) identifying the consequences of the users’ actions, so they can prevent future errors or make informed decisions about their practice; and (c) allowing users to customise information content delivered to them [44].

Considerable research has been undertaken into understanding the factors that enable health professionals to maintain clinical knowledge and competency [45–48]. It has been established that many adult learners value training and education that feels both authentic and is aligned with their experiences in the real world. The literature suggests that personal learning aligned to the learners’ experience is more impactful. For health professionals, the role of adaption has been noted through the Master Adaptive Learner (MAL) Framework [49], which can be used to guide skills acquisition by health professionals, with an emphasis on learners being adaptive in order to develop new clinical skills. The capacity of health professionals to act as adaptive learners was severely tested during the COVID pandemic [50]. Data captured within EHRs has a potential to support this learning adaptation. This is because the data could characterise different aspects of professional practice and by personalising learning for individual practitioners.

The physician feedback model (PFM) [51] describes the processes that health professionals use to reflect on their practice based on feedback and how they translate this into behaviour change. Although not designed to describe feedback derived from an EHR, the model provides an effective framing mechanism for understanding the process of reviewing EHR data, reflecting on practice and acting on that reflection if required. The PFM has three components to describe feedback to health professionals: (1) *reaction* — health professionals’ acceptance of clinical-performance feedback; (2) *action* — the behaviours health professionals engaging in after receiving feedback; and (3) *impact* — how a health professional translates feedback into patient-management behaviour. Consideration of the components of the PFM could help inform the design of EHR systems to support reflective practice and also support understanding of how health professionals might use EHR data. For EHR system design, the *reaction* component of the model suggests that effective feedback systems should contain data from a respected source, incorporate content that is relevant and present feedback in a timely/personalised manner. The *action* component of the model explains that health professionals will take three categories of action in response to feedback: (i) retroactive acts — revisiting previously seen patient interactions to correct a problem; (ii) proactive acts — focusing on changing interactions with future patients; and (iii) defensive acts — trying to justify performance. The first two acts are more conducive to a health professional accepting feedback, so it would be important to facilitate such reactions when using EHR data to give performance feedback. The *impact* component of the model suggests there are multiple factors that might influence whether a health professional changes their clinical practice behaviour based on feedback including their core values, emotional response and environmental factors.

The current design of EHRs does little to support such workplace-based learning by health professionals; ideal design of EHRs that would support learning is unclear. There are several key recommendations that if adopted could improve the utility of EHRs to support practice reflection and learning. A first recommendation would be for vendors to prioritise development and implementation of EHRs that are also tools for documenting health professional learning [40] and not just for documenting patient care [37]. A second recommendation would be for EHR designers to be guided by the principles of the effective feedback, such as those described in the PFM, as outlined above [51]. Designing such an approach is likely complex as it may need to account for issues related to data quality and completeness that are currently a challenge for the healthcare sector [52]. A third recommendation is to explore the application of machine learning to the plethora of structured and unstructured data in EHRs to help develop smarter profiles of health professional clinical practice and map such data to create individual clinical practice models to support learning.

It is likely that EHR design may need to scaffold people in using the data and to personalise workplace learning to be delivered when and in a way the individual is most receptive to change. Such scaffolding has potential to help health professionals understand and enhance their learning, as well as track and assess their learning progression over time. Promising scaffolding approaches include comparing current and historical performance to track performance changes over time, comparing performance against peers using metrics according to stage of training/experience, and using EHR data to compare performance against a defined standard [53]. Redesigned EHRs may automatically accumulate evidence that continuing professional development requirements have been achieved, a proposal that has have already been noted in the literature [40]. Coupled with this, designers of EHRs that support learners may need to think beyond the specific information system, to the healthcare context they are implemented in.

Whilst EHR design has a long way to go if it is to support learning, there is an equally large gap in understanding when and how health professionals want technology to help them to reflect on their clinical practice. The processes health professionals use to reflect on feedback are described in the PFM [51]. What is not well understood is the role of technology in supporting or disrupting the processes described in models such as the PFM. The importance of considering adoption enablers and barriers of such systems is highlighted in the mixed adoption by health professionals of EHR alerts [54]. Such alerts are intended to prompt reflective practice at the point of decision making. Considering both the technological and human factors will be critical for the repurposing of EHRs to support reflective practice.

## Conclusions

Addressing the suboptimal use of EHRs in supporting learning and reflective practice is important due to the disruption occurring in the health sector related to both the design of informatics technology and changes in policy about the use of routinely collected data to reflect on clinical practice [55]. Perhaps more importantly, this gap should be addressed urgently because EHR data has the potential to transform health professionals’ learning by strengthening processes that are known to improve clinical performance by health professionals and potentially reducing time spent on activities that do not change behaviour or improve care. EHRs that are designed to support professional learning have the potential to facilitate feedback of outcomes data to health professionals, so that they have access to objective information and patient narratives that can be used for self-evaluation of clinical performance [6]. Furthermore, EHR data has potential to link clinical outcomes with learning activities and guide health professionals towards training that is also likely to influence patients’ quality of care [56].

Given the considerable potential of EHRs that scaffold health professional learning, and the growth of open source and non-enterprise EHRs [57], why are there no examples of ones that do this? Is this a blind spot that today’s EHR vendors have thus far overlooked that will in future be remedied? Does the gap represent a complex design problem that no one has yet been able to address? What is the role of cost as a barrier to develop EHRs that truly support practitioner learning? It is likely that the solution lies in answering not one but all of these questions. Whilst the gap remains, there is a significant missed opportunity in the digital health sector: EHRs that support reflective practice and transform learning for health professionals.
